# Downregulation of *Brassica napus MYB69* (*BnMYB69*) increases biomass growth and disease susceptibility *via* remodeling phytohormone, chlorophyll, shikimate and lignin levels

**DOI:** 10.3389/fpls.2023.1157836

**Published:** 2023-03-29

**Authors:** Na Lin, Mu Wang, Jiayi Jiang, Qinyuan Zhou, Jiaming Yin, Jiana Li, Jianping Lian, Yufei Xue, Yourong Chai

**Affiliations:** ^1^ Integrative Science Center of Germplasm Creation in Western China (CHONGQING) Science City and Southwest University, College of Agronomy and Biotechnology, Southwest University, Chongqing, China; ^2^ Engineering Research Center of South Upland Agriculture, Ministry of Education, Academy of Agricultural Science, Southwest University, Chongqing, China

**Keywords:** *Brassica napus*, MYB69, shikimates, lignin, *Sclerotinia sclerotiorum*, IAA, chlorophyll, biomass

## Abstract

MYB transcription factors are major actors regulating plant development and adaptability. *Brassica napus* is a staple oil crop and is hampered by lodging and diseases. Here, four *B. napus MYB69* (*BnMYB69s*) genes were cloned and functionally characterized. They were dominantly expressed in stems during lignification. *BnMYB69* RNA interference (*BnMYB69i*) plants showed considerable changes in morphology, anatomy, metabolism and gene expression. Stem diameter, leaves, roots and total biomass were distinctly larger, but plant height was significantly reduced. Contents of lignin, cellulose and protopectin in stems were significantly reduced, accompanied with decrease in bending resistance and *Sclerotinia sclerotiorum* resistance. Anatomical detection observed perturbation in vascular and fiber differentiation in stems, but promotion in parenchyma growth, accompanied with changes in cell size and cell number. In shoots, contents of IAA, shikimates and proanthocyanidin were reduced, while contents of ABA, BL and leaf chlorophyll were increased. qRT-PCR revealed changes in multiple pathways of primary and secondary metabolisms. IAA treatment could recover many phenotypes and metabolisms of *BnMYB69i* plants. However, roots showed trends opposite to shoots in most cases, and *BnMYB69i* phenotypes were light-sensitive. Conclusively, BnMYB69s might be light-regulated positive regulators of shikimates-related metabolisms, and exert profound influences on various internal and external plant traits.

## Introduction

MYB is a large family of transcription factors (TFs) (Feller et al., 2011). It has been a hotspot in the study of the plant transcription factor function because of its large number of genes and different functions. The first identified MYB TF in plants was COLORED1 (C1), which is involved in the biosynthesis of anthocyanins in *Zea mays* (Paz-Ares et al., 1987). MYB TFs contain a highly conserved DNA binding domain (DBD) located at the N-terminus (Baldoni et al., 2015; Wei et al., 2017; Zhuang et al., 2021). The MYB domain is composed of 1-4 imperfect repeats (R1-R4) each containing approximately 50-53 amino acids (aa). Each repeat consists of three α-helices, of which the second and third ones form a helix-turn-helix (HTH) motif (Baldoni et al., 2015). The HTH intercalates with the major groove of DNA. MYB TFs have three spaced Trp residues forming a hydrophobic core that stabilizes the structure (Ogata et al., 1995). The C-terminus is responsible for the distinct regulatory activities of MYB TFs (Jin and Martin, 1999).

MYB TFs are divided into four groups, 1R-MYB, R2R3-MYB, 3R-MYB, and 4R-MYB, based on the number of repeats (Dubos et al., 2010). R2R3-MYB transcription factors (TFs) have been shown to play important roles in plants, including cell fate and identity, developmental processes, and responses to biotic and abiotic stresses (Hajiebrahimi et al., 2017). Although members of the *MYB* superfamily have been annotated in *Arabidopsis thaliana* and many other plants, most of them have not been functionally characterized.

In the past decade, *R2R3-MYB* genes have been extensively studied. The R2R3-MYB proteins play important roles in diverse biological processes including growth and development, primary and secondary metabolism such as flavonoid and anthocyanin biosynthesis as well as abiotic and biotic stress responses (Yao et al., 2020). Plant development and secondary metabolisms are related to cell differentiation including the change of cell size, cell number and cell wall. It was well known that the plant cell wall is a dynamic barrier against pathogens invasion (Underwood, 2012). The cell walls of vascular bundles deposit a large amount of cellulose, lignin and pectin, which are closely related to the support and resistance of plants. Lignin plays an important role in plant growth and development, lodging resistance and structural support (Jiang et al., 2020). The deposition of unpolymerized lignin monomers is considered to present a physical barrier that prevents infection in the interaction between plant and pathogen, and its metabolism is also actively involved in plant lodging resistance and in response to various environmental stresses (Jiang et al., 2020).

Many studies have shown that MYB69, which is from subgroup S21, is a SCW (secondary cell wall) activator (Kranz et al., 1998; Zhong et al., 2007; Dubos et al., 2010; Zhu et al., 2017). It has been demonstrated by transcriptional activation analysis that MYB69 and other transcription factors are able to activate the expression of the *HIS3* and *b-GAL* reporter genes in yeast, confirming that they are indeed transcriptional activators (Zhong et al., 2008). AtMYB69 is a positive regulator dedicated to cell wall thickening of fiber cells in *A. thaliana* (Dubos et al., 2010). Zhao and Bartley (2014) have shown that AtMYB69 is an activator whose dominant repression reduces SCW thickening of both interfascicular fibers and xylem fibers in stems. These studies indicate that *MYB69* may be related to the growth of plant cell. Studies have also shown by cluster analysis that *MYB105, MYB110, MYB117*, *MYB56*, *MYB52*, *MYB54* and *MYB69* belong to the same subgroup, so it is probable that their functions are similar (Kranz et al., 1998; Dubos et al., 2010; Zhu et al., 2017). *MYB20*, *MYB63*, *MYB69*, *SND2* and *SND3* are also hypothesized to be part of the SND1/NST1 transcriptional network regulating secondary wall biosynthesis (McCarthy et al., 2010). Xu et al. (2021) reported that *FvMYB3, FvMYB9, FvMYB11, FvMYB22, FvMYB64*, and *FvMYB105* mostly expressed at green stage of fruit development in woodland strawberry, aligned with proanthocyanidins accumulation. Wang et al. (2022) found that both *35S*::*MYB10* and *MYB110* can upregulate anthocyanin biosynthesis in *Actinidia chinensis* fruit. Hong et al. (2021) have identified MYB117 as a negative regulator in controlling flowering time through regulating the expression of *Flowering Locus T* in *Arabidopsis.* Other researchers showed that *SiMYB56* enhanced ABA synthesis under drought conditions and activated the ABA signaling pathway, contributing to the enhanced drought tolerance of transgenic rice (Xu et al., 2020). It has also been demonstrated that dominant repression of *MYB69* and *SND2*, *SND3*, *MYB103*, *MYB85*, *MYB52*, *MYB54*, and *KNAT7* causes a severe reduction in secondary wall thickening of both interfascicular fibers and xylary fibers in inflorescence stems (Zhong et al., 2008). It was reported that ERF4 and MYB52 transcription factors played antagonistic roles in regulating homogalacturonan de-methylesterification in *Arabidopsis* seed coat mucilage (Ding et al., 2021). For MYB46 downstream transcription factors, *MYB43, MYB52, MYB54, MYB58*, *MYB63* and *KNAT7* are direct targets of MYB46 (Zhong and Ye, 2012), which was a master transcriptional regulator of secondary wall biosynthesis (Kim et al., 2012). And it was reported that MYB46 is negatively regulated by MPK6 during salt stress (Im et al., 2021). These studies have revealed that the function of *MYB69* was not only related to regulating secondary wall biosynthesis but also probably related with some metabolisms and resistances in plant.

*B. napus* is an important oil crop worldwide and is negatively impacted by a variety of biotic and abiotic stresses, particularly lodging and *S. sclerotiorum* stem rot, which seriously reduce the yield and affect the quality of *B. napus* seeds. Lodging is related to stem and root lodging (Li and Qi, 2017; Zhang et al., 2021). The flexibility of stems and roots is related to secondary wall thickening and lignin accumulation. Studying the function of *MYB69* in *B. napus* can both elucidate whether it contributes to lodging resistance and antimicrobial resistance in *B. napus* and systematically analyze how *MYB69* regulates plant cell growth and development.

In this work, downregulation of *BnMYB69* causes an increase in the biomass and susceptibility to *S*. *sclerotiorum*, and related biochemical, physiological and gene expression characterization provides insight into *BnMYB69* function in *B. napus*.

## Materials and methods

### Plant materials

The material of gene cloning and expression characterization was cv. Zhongyou 821 (ZY821) of *B. napus.* The explant material of genetic transformation is cv. Zhongshuang 10 (ZS10) of *B. napus.* These plants were from growth chambers, green houses or isolated field farms at Southwest University and cultivated by standard agronomic management*. Nicotiana benthamiana* plants for protein localization study were cultured in room at 28°C under a 16 h/8 h light/dark cycle.

### Fungal strains

The strain of *S. sclerotiorum* in this experiment was provided by Dr. Jiaqin Mei at Southwest University. *S. sclerotiorum* was grown up on potato media (PDA) at 22°C with 85% humidity.

### Cloning of *BnMYB69*s

Total RNA was isolated from ZY821 organs using EASYspin plant RNA rapid extraction kit (Biomed, China). Total DNA was extracted from ZY821 leaves using traditional CTAB method. The quality and quantity of RNA and DNA were detected by agarose gel electrophoresis and NanoDrop 2000c (Thermo Fisher, USA).

In-silico cloning of *Brassica napus MYB69*s was performed on NCBI (https://blast.ncbi.nlm.nih.gov) using *A. thaliana MYB69* (*AtMYB69*) mRNA as Query in BLASTn against *B. napus* (taxid:3708) nr/nt, refseq_genomes, refseq_rna, est, and TSA databases. Sequences orthologous to *AtMYB69* were downloaded and multi-aligned on Vector NTI Advance 11.51, and primers for RACE (rapid-amplification of cDNA ends) cloning were designed (**Table S1**).

Using the SMARTer RACE Amplification Kit (Clontech, USA), 1 µg of total RNA mixture from various organs of ZY821 was used for synthesis of first-strand total cDNA. The primer pairs RBnMYB69-51 + LUPM and RBnMYB69-52 + NUP were used for the primary and the nested PCRs in 5’-RACE of *BnMYB69*s, while primer pairs FBnMYB69-31 + LUPM and FBnMYB69-32 + NUP were used for primary and nested PCRs in 3’-RACE of *BnMYB69*s. Based on sequencing results of 5’- and 3’-RACE colonies, primers FBnMYB69-1 + RBnMYB69-1, FBnMYB69-2 + RBnMYB69-2, FBnMYB69-3 + RBnMYB69-3 and FBnMYB69-4 + RBnMYB69-4 were used to amplify full-length cDNAs and gDNAs of *BnMYB69-1*, *BnMYB69-2*, *BnMYB69-3* and *BnMYB69-4*, respectively. Electrophoresis of PCR products, gel recovery, TA cloning, *Escherichia coli* transformation, colony culture and other experiments abided by routine methods. Bioinformatics Analyses of *BnMYB69*s genes and proteins were performed on local Vector NTI Advance 11.51 and websites of NCBI, Expasy, Softberry, etc. (See **Supplementary Tables and Figures** for more information).

### Quantitative RT-PCR (qRT-PCR) analysis

The total RNA of each organ or tissue sample from material was reverse transcribed by the PrimeScript 1st Strand cDNA Synthesis Kit (TaKaRa, Dalian, China) to obtain the corresponding first-strand total cDNA. All cDNA samples were diluted 20-fold using sterile water for qRT-PCR. The overall and member-specific expression levels of *B. napus* genes were detected by Gene-specific primers for qRT-PCR, respectively.

Primers are listed in **Table S1**, and the *25SrRNA* gene was used for the internal standard. The CFX Connect Real-Time PCR Detection System (Bio-Rad, Berkeley, CA, USA) with FastStart Universal SYBR Green Master reagents (Roche, Basel, Switzerland) was performed for qRT−PCR analysis. The program was that firstly, the sample was put in 95°C 10 min, followed by 45 cycles of amplification (95°C 10 s, 58-64°C 30 s). Secondly, the temperature was raised from 65 to 95°C, and the specificity of the amplification was confirmed by the melting curve. The detection was repeated three times. Thirdly, CFX Manager 3.1 (Bio-Rad, Berkeley, CA, USA) by the 2^-△△CT^ method analyzed all data.

### Subcellular localization

The coding sequence of *BnMYB69-1*(*BnaA01.MYB69*) was amplified using the primer pair FBnMYB69SL + RBnMYB69SL (**Table S1**), and it was cloned and designated *BnMYB69SL*. Then *BnMYB69SL* was subcloned into pEGAD by *Eco*RI+*Bam*HI double digestion, and enhanced green fluorescent protein (EGFP) was joined into the N-terminus to form the subcellular localization expression vector pEGAD-*BnMYB69SL* (**Figure S4A**). The vector was introduced in *Agrobacteriu tumefaciens* strain LBA4404 and transformed into tobacco leaves. Then the plant was cultured at 28°C for two days, stained its leaves with nuclear dye (DAPI) and photographed with a confocal microscope (LSM 800, ZEISS, Jena, Germany).

### RNAi vector construction and genetic transformation

Using the primer pair FBnMYB69I *+* RBnMYB69I (**Table S1**), a 429 bp C-terminus coding region of cDNA for the *BnMYB69* family (*BnMYB69s)* was amplified from the cDNA library. This cDNA fragment had >86.7% identity match with all *BnMYB69* genes, and no identity match with other sequences which were found by BLASTing the entire *B. napus* genome (http://www.ncbi.nlm.nih.gov/genome/?term=Brassica+napus). An antisense fragment of *BnMYB69I* (*BnMYB69IA*) was subcloned into pFGC5941M using *N*coI+*Aat*II double digestion to form the intermediate vector pFGC5941M-*BnMYB69IA* (**Figure S4B**). And a sense fragment of *BnMYB69I* (*BnMYB69IS*) was subcloned into pFGC5941M-*BnMYB69IA* using *Bam*HI+*Xba*I double digestion to form the RNAi vector pFGC5941M-*BnMYB69I*. *CaMV35S* was the promoter of the vector. pFGC5941M-*BnMYB69I* plasmids were introduced into LBA4404 *(A. tumefaciens)* by the freeze-thaw transformation method. The engineering strain harboring pFGC5941M-*BnMYB69I* was transformed into hypocotyl segments of ZS10 using the method described by Cardoza and Stewart (2003). The positive transgenic plants were detected by DNA electrophoresis after PCR amplification (**Figure S4C**).

### Measurement of agronomic traits

Transgenic and nontransgenic *B. napus* (WT) plants were grown in a greenhouse, agronomic traits were investigated at the bolting stage, full flowering stage and pod setting stage, and dry matter weight, chlorophyll content, and 1000 seed weight per plant were measured at the harvest stage.

### Assessment of resistance to *S. sclerotiorum*

The method of leaf inoculation was from Godoy et al. (1990). The third or fourth leaf from the top in each plant at the 9 to 12-leaf stage was excised with at least 6 leaves per different line. The excisions of these leaves were wrapped with wet paper to keep moisture, and were placed into porcelain plate paved with wet filter paper. 7-mm-diameter PDA discs containing *S. sclerotiorum* hyphae were inoculated at the center of the left and right abdomens in leaf. Then, the whole plate was covered with cling film and kept at 22°C. After 48 h, the long diameter (a) and the short diameter (b) of the lesion were measured. The lesion area was gained by this calculation formula: S = (π×a×b)/4.

The method of stem inoculation was from Mei et al. (2015). The stems were inoculated at the full-bloom stage with 6 plants per different line. Stem fragments (45 cm long) were cut off at 20 cm from the ground, and both ends also were wrapped with wet paper and covered with cling film. 7-mm-diameter PDA discs containing *S. sclerotiorum* hyphae were inoculated at two points distanced with 10 cm on the stem segment. Lesion lengths were measured after 72 h.

During *S. sclerotiorum* infection, leaves were taken at different time points for the determination of relevant biochemical components and key enzymes, and RNA was extracted for qRT−PCR (as described above).

### Microscopic observation of tissue section

The methods of Mäule staining, phloroglucinol-HCl staining and fluorescent brightener dyeing of lignin were from the literature (Zhong et al., 2007; Yin et al., 2017). Cellulose staining with Fast Green FCF and pectin staining by the hydroxylamine method (Leagene, Beijing, China) were performed on the stems and roots of transgenic and WT plants. Stained sections were observed with a stereo light microscope (Olympus SZX2-FOA, Tokyo, Japan) and a fluorescence microscope (Nikon Eclipse E600 W, Tokyo, Japan). The tissues and organs were observed by scanning electron microscopy (Hitachi SU3500, Japan).

### Determination of cell wall and primary metabolism composition

At the harvest stage, the middle part of mature stems was cut off and dried at 70°C to constant weight. Then, these stems were powdered with a grinder, and screened with a 100-mesh sieve. The cellulose, lignin and pectin contents from the cell walls of transgenic and WT plants were extracted and determined. The method of extraction and determination of cellulose and lignin was from Foster et al. (Foster et al., 1996). The method of extraction and quantification of pectin was from Blumenkrantz and Asboe-Hansen (1973). The absorbance of cellulose and lignin was determined by an Infinite M2000 Pro Microplate Reader (Tecan, Männedorf, Switzerland). The contents protopectin and soluble pectin were determined according to the instructions of the Protopectin and Soluble Pectin Content Determination kits from Shanghai Haling Biotechnology Co., Ltd.

At bolting stage, the middle-part samples of stems, leaves and roots were fetched and stored in a -80°C freezer. The frozen samples were ground into powder with liquid nitrogen to test the activity of CAT, MDA, SOD and POD, as well as the contents of chlorophyll, reducing sugars, and total amino acids. The extraction and determination of these biochemical components followed the kit instructions from Beijing Leagene Biotech Co., Ltd.

### Determination of hormone components

At the bolting stage, the mid-region fragments of stems and leaves were sampled and stored in a -80°C refrigerator. Frozen samples were sent to Qingdao Kechuang Quality Inspection Company and Nanjing Zhongding Biotechnology Co., Ltd. to detect the changes in hormone components using gas chromatography or liquid chromatography, respectively.

### Hormone treatment

Cultured in 1/3 X Hoagland nutrient solution, WT and *BnMYB69i* lines were treated with 100 µmol/L IAA, with non-addition as the experimental control (CK). They were cultured on Petri dishes (light 16 h/22°C; dark 8 h/18°C) and then in large pots after two weeks with 40 seedlings per pot. The water was changed every 10 days. There were 2 lines, 2 treatments and 3 repetitions. The morphological, physiological and biochemical indices were measured at the cotyledon stage (20 days), 3 true leaves stage (60 days) and 5 true leaves stage (120 days). Three plants were randomly selected from each line for the measurement of plant height, stem diameter and physiological and biochemical indices, and the average values of each parameter were calculated. Plant height was defined as the length from the cotyledonary node to the highest part of the entire plant (Gai, 2005); stem diameter of the stem base at the cotyledon mark was measured with a vernier caliper.

WT and transgenic plants were cultured in high light (8000 lx) and low light (4000 lx) incubators. They grew to the three-leaf stage under light for 16 h at 22°C and under darkness for 8 h at 18°C, and the chlorophyll content of the third true leaf was measured. The methods were performed according to instructions of the Beijing Leagene Biotechnology kit and the experimental guidance of Plant Physiology handbook (College of Agriculture/Department of Plant Physiology, Shanxi Agricultural University).

The transgenic and control plants were treated in the dark three times for 20 min each at different stages. The chlorophyll fluorescence kinetic parameters were determined with a Spectral Fluorescence Imager and Fluor Scan software.

A 0.1 g sample of *B. napus* leaves was frozen in liquid nitrogen and 300 μL of 1% hydrochloric acid methanol solution was added after proofing, mixed evenly and shaded at 4°C overnight. The next day, 200 μL distilled water was added along with 200μL chloroform, the mixture was vortexed for 20 s at room temperature and then centrifuged for 5 min at 17000 g. The extracted supernatant was added into a 96-well plate and determined using an Enzyme Labeling Instrument at 530 nm and 657 nm. The proanthocyanidin content (mg/g) was calculated as (A530-A657)/fresh weight (g).

The IAA content was determined according to the instructions of the plant indole-3-acetic acid ELISA kit (Nanjing Camilo Bioengineering Co., Ltd.), and the method of quantifying protopectin and amino acids was as described above.

### Statistical analysis

In this study, at least three biological replications (n≥3) were performed for each experiment. All values are presented as the mean ± standard erro (SE). Asterisks indicate significant or extremely significant differences from WT (*0.01≤ p < 0.05, ** for p < 0.01) using a one-way ANOVA.

## Results

### Characterization of *MYB69s* from *B. napus*

Using mixture total RNA from various organs of ZY821 as start material, our RACE-PCR based cloning isolated four *MYB69* genes from *B. napus*, i.e., *BnMYB69-1 (BnaA01.MYB69)*, *BnMYB69-2 (BnaC01.MYB69)*, *BnMYB69-3 (BnaA08.MYB69)* and *BnMYB69-4 (BnaC03.MYB69)*, with respective gene lengths of 1110, 1418, 1296 and 1215 bp, mRNA lengths (not including polyA) of 1015, 1320, 1191 and 1110 bp, and coding region (open reading frame, ORF, including stop codon) lengths of 756, 753, 732, and 756 bp, respectively. *BnMYB69-1*, *BnMYB69-2*, *BnMYB69-3* and *BnMYB69-4* showed high homologies to *AtMYB69*, with gene level identities of 84.2%, 78.3%, 77.8% and 80.0%, mRNA level identities of 85.5%, 80.0%, 79.5% and 80.8%, and ORF level identities of 86.5%, 86.2%, 84.8 and 87.0%, respectively (**Table S2**). Only one intron exists in each of the four *BnMYB69* genes. 5’UTR length of *BnMYB69* genes is 28-132 bp, and their 3’UTR length is 127-494 bp. Every *BnMYB69* gene shows alternative transcription start sites (TSS) and alternative polyadenylation sites, thus multiple versions of mRNAs could be cloned with differed length of 5’UTRs and 3’UTRs. One feature is that in most cases the TSS base is a purine (A or G), and in most cases the base before the polyA tail is a pyrimidine (T or C). Another feature is that as *BnMYB69-1* could generate long 5’UTRs, its 3’UTRs are relatively shorter, while the other three *BnMYB69* genes have the opposite trend (**Table S2**).

The predicted BnMYB69-1, BnMYB69-2, BnMYB69-3, BnMYB69-4 proteins have a length of 251, 250, 243, 251 aa, a MW of 29.01, 28.90, 28.16, 29.04 kD, and a *pI* of 9.58, 9.70, 9.72, 9.56 implying that they are basic proteins. All of them have no signal peptide and no transmembrane helix, and are predicted to be located in the nuclear. Their predicted phosphorylation sites are 19-22 for S (serine), 8-9 for T (threonine), and 1-2 for Y (tyrosine). The N-terminal half of each BnMYB69 protein has two predicted HTH MYB-type domain regions at 14-65/64 and 66-120, on which there are two HTH motif DNA-binding regions at 42-63 and 93-116 (**Table S3**). The front HTH model belongs to the R2 subdomain and has three conserved tryptophan residues (W), which are separated by 18 or 19 amino acids, and the latter HTH model belongs to the R3 subdomain (Ogata et al., 1994). In the secondary structure of BnMYB69s, α-helix, extended strand, β-turn, and random coil occupy 23.11%-31.87%, 6.77%-12.75%, 4.38%-6.40%, and 54.40%-59.76%, respectively (**Figure S1**). SWISS-MODEL-predicted tertiary structures of BnMYB69s conform to conserved R2R3-MYB domain (**Figure S2**). Thus, the four *BnMYB69* genes cloned here most probably encode typical R2R3-MYB transcription factors.

**Figure 1:**
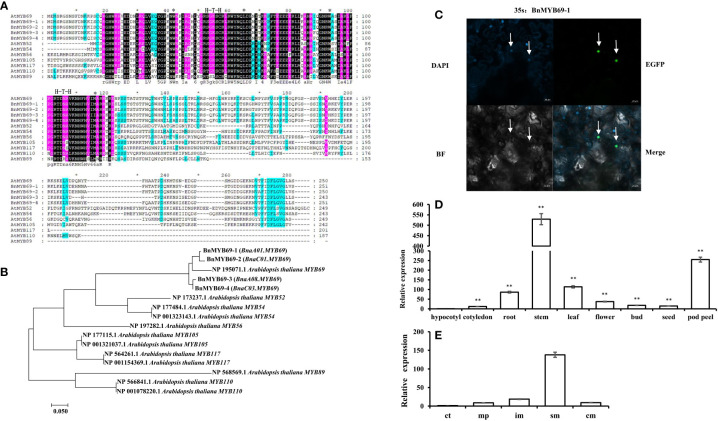
Cloning and analysis of *BMYB69s.* **(A)** Sequence alignment analysis of AtMYB69 and S21*-*AtMYB proteins. (*Between them are amino acid residues with H-T-H motif structure) **(B)** Molecular phylogenetic analysis by maximum likelihood method of BnMYB69s with S21 AtMYBs. **(C)** Subcellular localization of BnMYB69-1. (The arrow refers to the nucleus) **(D)** *BnMYB69s* overall expression in different organs of *B. napus*. **(E)** Expression of *BnMYB69s* in different parts of the main stem of *B. napus*. Note, (ct) growth cone tip. (mp) main-stem primordium. (im) initially lignified main-stem, (sm) semi-lignified main-stem. (cm) completely lignified main-stem. At least three biological replications (n≥3) were conducted for each experiment. All values are presented as the mean ± standard deviation (SD). Asterisks indicate significant or extremely significant differences from the first series (*0.01<p < 0.05; **p< 0.01) using one-way ANOVA.

In multiple alignment, BnMYB69 proteins have high similarities to AtMYB69 on the whole protein level. Though they show high similarities to MYB52, MYB54, MYB56, MYB105, MYB117, MYB110 and MYB89 of S21 in the N-terminal half, the similarities in the C-terminal half are distinctly lower with conservation only at a C-terminal motif and several dispersed residues (**Figure 1A**). Phylogenetic analysis showed that the distances among BnMYB69 proteins are small, and they clustered tightly with AtMYB69. BnMYB69-1 and BnMYB69-2 are close, while BnMYB69-3 and BnMYB69-4 are close, but these two sister pairs are a little far from each other. In the tree, the distances of BnMYB69s to other S21 AtMYB proteins are much larger than to AtMYB69 (**Figure 1B**). These results clearly show that all *BnMYB69* genes isolated here belong to S21 MYBs and are orthologous genes of *AtMYB69*.

To confirm the subcellular localization of BnMYB69s, experimental validation was carried out. Consistent with the bioinformatics prediction and its predicted role as a transcription factor, the expression of enhanced green fluorescent protein (EGFP)-labeled BnMYB69-1 and blue fluorescent nucleic acid dye (DAPI) coincided in tobacco leaves, showing that they were both localized in the nucleus (**Figure 1C**).

In qRT-PCR results, overall *BnMYB69* expression could be detected in all organs of *B. napus* but varied greatly among them. It was highly expressed in stems, moderately expressed in pod peels, leaves and roots, and lowly expressed in seeds, flowers, buds, cotyledons, and hypocotyls (**Figure 1D**). At bolting stage, different stem sections were detected for *BnMYB69* expression, which showed an increase trend in parallel with the stem vascularization/lignification process, with little expression in growth cone tip and highest expression in semi-lignified main-stem. However, its expression in the completely lignified main-stem dropped down (**Figure 1E**). We also detected the organ-specificity of the four individual member genes, and all of them showed typical stem-specific expression patterns (**Figure S3**). Overlapping result of the four genes’ expression patterns was not perfectly coincided to the overall expression pattern, suggesting that there might be other member genes that have not been cloned in this study. We further checked the BLAST results, and found that the *B. napus* genome might contain another two *BnMYB69* genes beyond these four genes.

### Silencing of *BnMYB69* increased biomass and altered chlorophyll and phytohormone profiles

**Figure 2:**
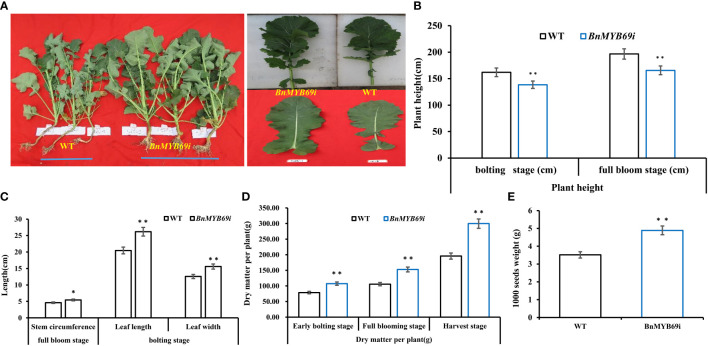
Morphological observation and dry matter weight of transgenic and WT plants. **(A)** Comparison of plant morphology between transgenic and WT plants in each generation. **(B)** Comparison of plant height of transgenic and WT plants at different stages. **(C)** Comparison of stem circumference and leaf of transgenic and WT plants. **(D)** Comparison of dry matter weight of transgenic and WT plants at three growth stages. **(E)** Comparison of 1000 seeds weight of transgenic and WT plants. Note, (WT) wild type *Brassica napus* cv. Zhongshuang 10 (BnMYB69i) *BnMYB69* RNA interference rapeseed (the same below). Asterisks indicate significant or extremely significant differences from WT.

**Figure 3:**
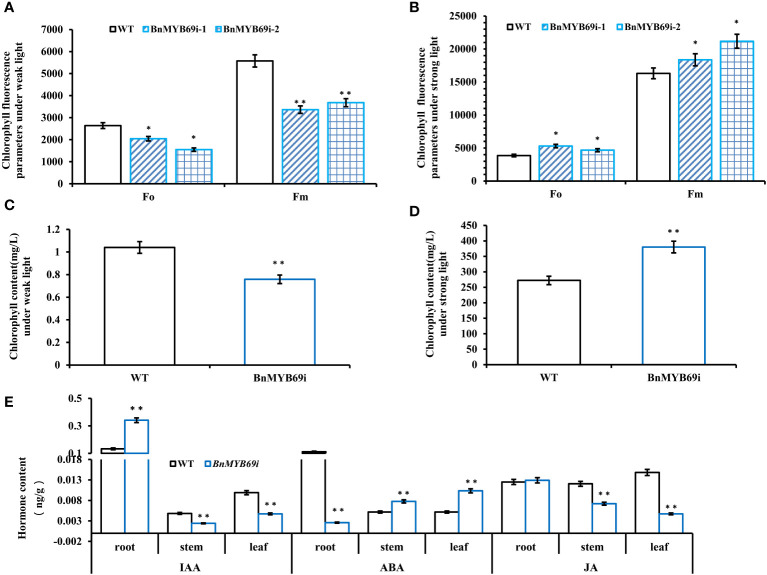
Analysis of chlorophyll content in different materials under different light intensities. **(A)** Chlorophyll fluorescence parameters under weak light. **(B)** Chlorophyll fluorescence parameters under strong light. **(C)** Chlorophyll content under weak light. **(D)** Chlorophyll content under strong light. **(E)** Comparison of three hormones in different organs between transgenic and WT plants. (root) hormone content of root. (stem) hormone content of stem. (leaf) hormone content of leaf. Asterisks indicate significant or extremely significant differences from WT (*0.01≤p < 0.05; **p < 0.01) using one-way ANOVA.

Compared with the WT plants, the *BnMYB69i* plants had larger leaves, a higher chlorophyll content, heavier 1000-seed weight and shorter height when grown in the field (**Figures 2**, **3**). The leaf area of transgenic plants at the bolting stage was significantly larger than that of the wild type (**Figure 2C**). The dry weight of *BnMYB69i* plants at the early bolting stage, full bloom stage and harvest stage increased by 36.4%, 44.5% and 53.2%, respectively, compared with those of the WT (**Figure 2D**). The 1000 seed weight of transgenic plants increased by 7.35% to 55.57% compared with that of WT plants (**Figure 2E**).

The chlorophyll fluorescence analysis showed that *BnMYB69*-silenced plants were very sensitive to light intensity (**Figures 3A, B**). Furthermore, the analysis of the chlorophyll content of *BnMYB69i* plants under different light conditions showed that chlorophyll synthesis in *BnMYB69i* plants may be related to light intensity (**Figures 3C, D**).

The hormone auxin (IAA) and 6-deoxocastasterone (6DS) in stems decreased significantly (**Tables 1A**, **B**); in contrast, abscisic acid (ABA) and brassinolide (BL) increased significantly (**Tables 1A**, **B**). In addition, IAA decreased and ABA increased in both stems and leaves, but the opposite was true in roots (**Figure 3E**). These results may explain the changes in morphological characteristics of transgenic plants.

**Table 1A:** Comparison of thirteen hormones in stems of transgenic and WT plants.

Material	Plant hormones (ng/g)						
	IAA	ABA	IBA	GA1	GA3	GA4	GA7
WT	19.81 ± 0.9892	5.69 ± 1.2799	0.05 ± 0.0070	0.07 ± 0.0014	0.07 ± 0.0131	0.11 ± 0.0267	0.06 ± 0.0114
*BnMYB69i*	7.19 ± 0.7958**	24.56 ± 3.1883**	0.12 ± 0.0318	0.12 ± 0.0219	0.05 ± 0.0057	0.23 ± 0.0652	0.03 ± 0.0168
Material	TZR	IP	IPA	SA	JA	MeJA	
WT	0.53 ± 0.1228	0.02 ± 0.0031	0.62 ± 0.0861	12.97 ± 2.2208	23.57 ± 2.0096	0.12 ± 0.0073	
*BnMYB69i*	0.82 ± 0.1236	0.01 ± 0.0032	0.97 ± 0.1517	9.36 ± 0.4046	23.73 ± 2.9764	0.28 ± 0.0641	

IAA, indole-3-acetic acid (auxin); ABA, Abscisic acid; IBA, indolylbutyric acid; GA1, gibberellin 1; GA3, gibberellin3; GA4, gibberellin 4; GA7, gibberellin 7; TZR, Trans Zeatin Riboside; IP, indolepro pionic acid; SA, Salicylic acid; JA, Jasmonic acid; MeJA, methyl jasmonate.

**Table 1B:** Comparison of three hormones in transgenic and WT plants.

Material	Rapeseed hormones (ng/g)		
	BL	6DS	CS
WT	0.04 ± 0.0057	186.73 ± 12.8313	1.05 ± 0.1266
BnMYB69i	0.19 ± 0.0233**	123.83 ± 3.5775**	1.09 ± 0.0926

BL, brassinolide; 6DS, 6-deoxocastasterone; CS, castasterone. Because WT was too small, the contents of rapeseed hormone strigolactone (5DS) was not determined. (WT) ZS10 inbred pure material (explant of transgenic study). (BnMYB69i) Transgenic plants with the third generation of selfing. All values are presented as the mean ±standard error (SE) (n=3).

### Silencing of *BnMYB69* impaired vascular and fiber development, secondary wall lignification, and mechanical strength of the stem

**Figure 4:**
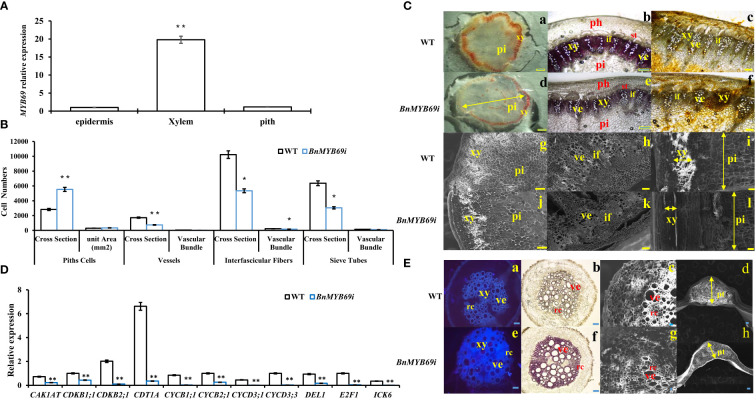
*BnMYB6*9s Expression and analysis in stem tissues and microscopic observation of stem and root. **(A)** Expression of *BnMYB69* in different parts of main stem of rapeseed. **(B)** Number of cells of piths, sclerenchyma, vessels, interfascicular fibers and sieve tubes of stems cross-section of *BnMYB69I* transgenic plants. Cross Section is the whole cross section for Pith Cells, and is a microscopic vision of the cross section for Vessels, Interfascicular Fibers and Sieve Tubes. **(C)** Histochemical staining and electron microscopic observation of cross-section of stem. **(a-c)** Histochemical staining of stems of WT. **(d-f)** Histochemical staining of stems of *BnMYB69* silenced plants. **(a, d)** Mäule staining of lignin in whole cross-sections. **(b, e)** Phloroglucinol-HCl staining of lignin in partial cross-sections. **(c, f)** Hydroxylammonium staining of pectin in partial cross-sections. **(g-h)** Electron microscopic cross-section of stems of WT. **(i)** Electron microscopic longitudinal section of stem of WT. **(j-k)** Electron microscopic cross-section of stems of *BnMYB69* silenced plants, **(l)** Electron microscopic longitudinal section of stem of *BnMYB69* silenced plants. pi, Piths; xy, Xylem; ph, Phloem; ve, Vessel; if, Interfascicular fibers; st, Sieve tube. Bar, 2 mm **(a,d)**, 1 mm **(b, c, e, f, g-l)**. **(D)** The cell cycle related genes expression in stems of transgenic plant and WT. **(E)** Comparison of root, petiole and leaf morphology between transgenic and WT. **(a, e)** Fluorescent brightener dyeing of lignin in whole root cross-sections, **(b, f)** Phloroglucinol-HCl staining of lignin in whole root cross-sections, **(c, g)** Electron microscopic observation of root partial cross-sections, **(d, h)** Electron microscopic observation of petiole cross-section of transgenic and control plants. xy, Xylem; ve, Vessel; rc, Ray cell; pt, Petiole thickness. Bar, 1 mm **(a, b, e, f)**, 200 μm **(c, g)**,1 mm **(d, h)**. Asterisks indicate significant or extremely significant differences from WT (*0.01≤ p < 0.05; **p< 0.01) using one-way ANOVA.

*BnMYB69* is mainly expressed in vascular organs, particularly xylem (**Figure 4A**). *BnMYB69i* plants showed a phenotypic change related to vascular organ growth retardation (**Figure 4C**). The stem circumference of the transgenic plant was significantly larger than that of the WT (**Figure 2C**), and the staining of xylem and the vascular bundle showed that the total number of xylem and vascular bundles in the cross-section of the stem increased (**Figures 4Cb, c, e, f**), but the number per unit area decreased, and this structural change caused the stem to become loose and soft (**Figures 4B, C**). The xylem in the longitudinal section of the stem decreased significantly, and the width was only 30% of that of the WT (**Figures 4B, Ci, l**). The number of pith cells increased, and the number per unit area increased both horizontally and vertically (**Figures 4Ca, d, i, l**). In addition, compared with the WT, the root xylem of transgenic plants was stained deeper, indicating that the lignin content was increased (**Figure 4E**).

Statistical analysis showed that the number of pith cells in the unit area of transgenic plant stems increased by 14%, and the size and number of cells in cross section changed significantly (**Figure 4B**). In transgenic plants, the single xylem layer was loose and was approximately 43% thinner than that in the WT (**Figure 4B**), and the number of vessels in the vascular bundle of a microscopic vision of the cross section was also reduced by approximately 57% (**Figure 4B**). More significantly, the number of interfascicular fibers of transgenic plants in the cross-section decreased by 47.7%, and the number of interfascicular fibers between two vascular bundles decreased by 30% (**Figure 4B**). In addition, the number of pith cells in the cross-section of stem of transgenic plants increased extremely significantly by 95.6% (**Figure 4B**). The total number of vascular bundles in a microscopic vision of the cross section decreased significantly by 50.1% (**Figure 4B**). Furthermore, the sieve tube number in a microscopic vision of the cross section also decreased significantly in *BnMYB69i* plants (**Figure 4B**). Due to the changes in the number and size of xylem and pith cells in the cross-section of the stem, the cell cycle-related genes of the stem were analyzed by RT-PCR. The cell cycle-related genes *CAK1AT*, *CDKB1*;*1*, *CDKB2*;*1*, *CDT1A*, *CYCB1*;*1*, *CYCB2*;*1*, *CYCD2*;*1*, *CYCD3*;*1*, *CYCD3*;*3*, *DEL1*, *E2F1* and *ICK6* were significantly downregulated (**Figure 4D**).

**Figure 5:**
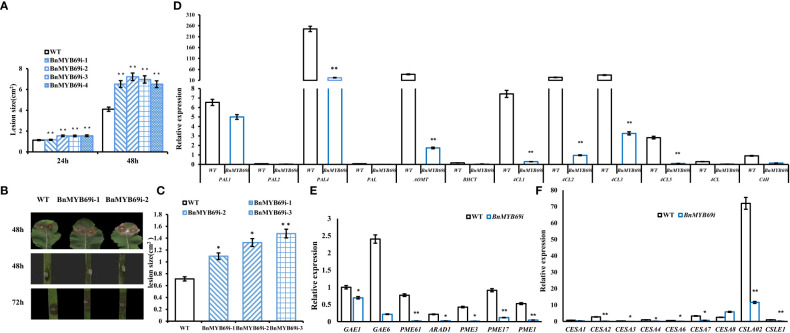
Expression of *BnMYB69*s and secondary wall biosynthesis genes in *B. napus* leaf and stem after *S. sclerotiorum* inoculation. **(A)** Statistics of leaf lesion area at different times after inoculation. **(B)** Lesion plaques on leaves and stems after *S. sclerotiorum* inoculation. **(C)** Statistics of stem lesion area at 48h after inoculation. **(D)** Lignin biosynthesis related genes expression in stems of transgenic plant and WT. **(E)** Pectin biosynthesis related genes expression in stems of transgenic plant and WT. **(F)** Cellulose biosynthesis related genes expression in stems of transgenic plant and WT. Asterisks indicate significant or extremely significant differences from WT (*0.01≤ p < 0.05; **p< 0.01) using one-way ANOVA.

Because of the changes in the stem xylem of transgenic plants, the resistance to *S. sclerotiorum* and stem strength were tested. After *S. sclerotiorum* inoculation, *BnMYB69* expression was upregulated at 3 and 9 h, distinctly upregulated at 24 h, and returned to a slightly higher than base level at 48 h, showing a dynamic change during the interaction between *B. napus* and *S. sclerotiorum* (**Figure 5**). This implies that the *BnMYB69* gene family may be involved in the interaction with *S. sclerotiorum* in oilseed rape. *BnMYB69i* plants were evaluated for decreasing *S. sclerotiorum* resistance. The stem and leaf lesion sizes were increased by 81.94% and 65.91%, respectively, compared with those of the WT, implying a substantial decrease in resistance to *S. sclerotiorum* stem rot disease (**Figures 5B, C**). It is clear that the suppression of *BnMYB69* significantly but solely weakened the resistance to *S. sclerotiorum* in vascular tissues in which innate *BnMYB69* is dominantly expressed.

In addition, the breaking resistance of the upper, middle and lower stems of transgenic plants was decreased by 47.31%, 11.93% and 37.65%, respectively, with an average value of 32.3% for the entire stem (**Table 2**), which indicates that the stem is fragile and the lodging resistance is reduced. In the stems of transgenic plants, compared with those of WT plants, the contents of neutral detergent fiber (NDF), acid detergent fiber (ADF) and lignin (ADL) decreased significantly by 17.17%, 13.48% and 30.04%, respectively (**Table 3**), whereas the contents of protopectin and soluble pectin decreased by 19.75% and 42.4%, respectively (**Table 4**). There was no significant difference in the pectin content of leaves between transgenic plants and WT plants (**Table 4**). In contrast, in the roots of transgenic plants, the contents of neutral detergent fiber (NDF), acid detergent fiber (ADF) and lignin (ADL) were significantly increased by 10.8%, 11.5%, and 9.3%, respectively (**Table 3**).

**Table 2:** Comparison of bending-resistance between transgenic and WT plants.

Material	Bending resistance (N)		
	Lower stem	Middle stem	Upper stem
WT	291.1±19.23	111.1±3.45	61.7±4.89
*BnMYB69i*	183.0±8.87**	90.8±4.54**	30.7±2.25**

Asterisks indicate significant or extremely significant differences from WT (*0.01≤ p < 0.05; **p< 0.01) using one-way ANOVA.

**Table 3:** Cell wall ingredients determination in stem and root by NIRs.

Material	Stem CWR (%)			
	NDF	ADF	ADL	S / G ratio
WT	71.25±1.45	56.05±0.55	12.70±0.46	0.351±0.001
*BnMYB69i*	66.70±0.51^*^	49.41±0.05^*^	8.90±0.02^**^	0.377±0.009^*^
Material	Root CWR (%)			
	NDF	ADF	ADL	S / G ratio
WT	76.02±0.30	>57.15±0.83	13.02±0.19	0.343±0.0012
*BnMYB69i*	84.25±2.47^*^	63.72±1.77^*^	14.22±0.10^**^	0.335±0.0015^*^

NDF, Neutral detergent fiber; ADF, Acid detergent fiber; ADL, Acid detergent lignin; S/G, syringyl(S)/guaiacyl (G) ratio. Asterisks indicate significant or extremely significant differences from WT (*0.01≤ p < 0.05; **p< 0.01) using one-way ANOVA.

**Table 4:** Protopectin content determination in stem and leaf.

Material	Protopectin (µmol/g)		Soluble pectin (µmol/g)	
	stem	leaf	stem	leaf
WT	171.31±10.14	264.31±2.01	79.50±20.03	56.18±7.29
*BnMYB69i*	137.64±3.86^*^	256.25±1.15	45.86±22.03^*^	60.86±2.87

Asterisks indicate significant or extremely significant differences from WT (*0.01≤ p < 0.05; **p< 0.01) using one-way ANOVA.

Rapeseed stems mainly contain S and G lignin types, and we found that the ratio of S/G increased significantly by 7.35% (**Table 3**), implying G lignin biosynthesis was more serviously impaired than S lignin biosynthesis. However, the S/G ratio of roots in transgenic plants decreased significantly, implying a trend opposite to the stem. These results showed that the silencing of *BnMYB69* significantly reduced the differentiation of lignification tissues, the deposition of stem secondary cell wall ingredients (such as lignin, cellulose and protopectin), and the mechanical strength in the stem, with little influence on leaf lignification and an increase in root lignification (**Table 3**).

The lignin biosynthesis-related genes *PAL1*, *PAL2, PAL4, PAL*, *C4H*, *4CL1*, *4CL2*, *4CL3*, *4CL5*, *4CL*, *COMT*, and *HCT* were downregulated in the stems of transgenic plants (**Figure 5D**). The pectin biosynthesis-related genes *GAE1*, *GAE6*, and *ARAD1* were significantly downregulated. Additionally, the pectin methylesterase genes *PME1*, *PME3*, *PME17*, and *PME61* were significantly downregulated (**Figure 5E**). In transgenic plants, the genes *CESA1*, *CESA2*, *CESA3*, *CESA4*, *CESA6*, *CESA7*, *CSLE1* and *CSLA02*, which are related to cellulose biosynthesis, were significantly downregulated (**Figure 5F**), and only *CESA8* was upregulated. The results showed that *BnMYB69* regulated the synthesis of lignin, cellulose and pectin, resulting in changes in breaking resistance and disease resistance.

### IAA treatment of transgenic *BnMYB69i* plants recovered many phenotypes and increased chlorophyll synthesis

**Figure 6:**
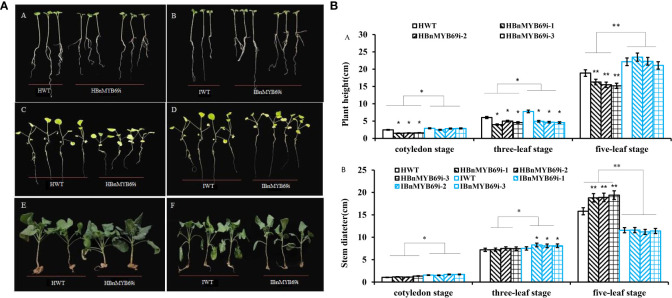
Phenotypic observation of transgenic plants and WT treated with H_2_O and IAA. **(A)** Growth of *BnMYB69* transgenic plants and WT under different treatments at different stages. **(A, B)** cotyledon stage. **(C, D)** three-leaf stage. **(E, F)** five-leaf stage. HWT, hydroponic WT plant; HBnMYB69i, hydroponic *BnMYB69* interfering plant; IWT, WT plant with IAA treatment; IBnMYB69i, *BnMYB69* interfering plant with IAA treatment (the same below). **(B)** Plant height and stem diameter of RNAi plants and WT under different treatments at different periods. **(A)** Change of plant height. **(B)** Change of stem diameter. Asterisks indicate significant or extremely significant differences from WT (*0.01≤ p < 0.05; **p< 0.01) using one-way ANOVA.

Phenotypic observation of the growth changes of *BnMYB69i* plants at the cotyledon stage and the three- and five-leaf stages showed that IAA treatment significantly increased the plant height of transgenic plants and inhibited the increase in stem circumference. The *BnMYB69i* plants recovered to the same size as the WT plants at the seedling stage in the growth chamber (**Figures 6A, B**).

**Figure 7:**
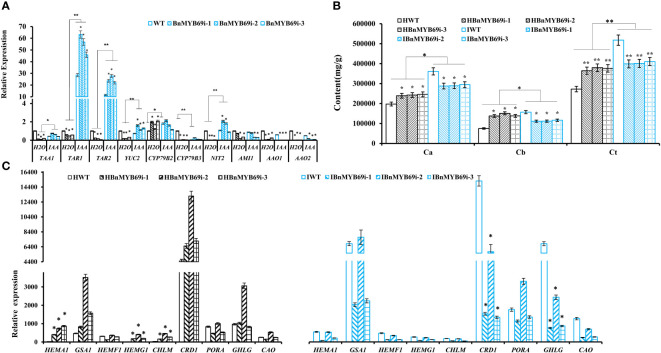
Changes of IAA and chlorophyll synthesis in transgenic and WT plants under different treatments. **(A)** Expression analysis of genes related to IAA synthesis pathway in transgenic and WT plants under different treatment. **(B)** Analysis of chlorophyll content difference of *BnMYB69* transgenic plants under different treatments. Ca, chlorophyll a; Cb, chlorophyll b; Ct, total chlorophyll. **(C)** Differential expression of key enzymes in chlorophyll synthesis pathway under different treatments. (H_2_O) Control plant with water instead of IAA. (IAA) plant with IAA treatment (the same below). Asterisks indicate significant or extremely significant differences from WT (*0.01≤ p < 0.05; **p< 0.01) using one-way ANOVA.

qRT-PCR analysis of the expression of key genes in the IAA synthesis pathway showed that the expression of the *TAA1*, *YUC2*, *TAR1*, *TAR2*, *CYP79B3*, *NIT2*, *AMI1*, *AAO1* and *AAO2* genes decreased significantly in the untreated *BnMYB69i* plants (P < 0.05), while the expression of *CYP79B2* increased significantly (P < 0.05). The expression levels of *TAR1*, *TAR2*, *YUC2*, and *NIT2* in *BnMYB69i* plants treated with IAA were significantly higher than those in untreated plants, but there was no significant difference in *TAA1, CYP79B2*, or *CYP79B3*. *AMI1*, *AAO1*, and *AAO2* were significantly lower than in untreated plants (P < 0.05) (**Figure 7A**).

The chlorophyll content of various lines under strong light was determined. The results showed that the total chlorophyll (Ct) of *BnMYB69i* plants increased significantly under strong light with an increase mainly in leaf chlorophyll a and a decrease in chlorophyll b (**Figure 7B**).

The expression of chlorophyll synthesis-related genes under IAA treatment was checked with qRT-PCR (**Figure 7C**). The expression levels of *HEMA1*, *HEMG1*, *CHLM*, *CRD1*, and *CHLG* in transgenic plants were significantly downregulated after treatment (P < 0.05), but the expression levels of *GSA1*, *PORA* and *CAO* were significantly upregulated in the transgenic plants (P < 0.05) (**Figure 7C**). There was no significant difference in *HEMF1* gene expression (**Figure 7C**). It was also shown that *BnMYB69* probably regulates the gene expression of *HEMA1*, *HEMG1*, *CHLM*, *CRD1*, and *CHLG* in some way.

### Silencing of *BnMYB69* affected shikimic acid metabolism and flavonoid-proanthocyanidin biosynthesis

Our above results indicated that *BnMYB69* is involved in the regulation of various metabolites, such as lignin, cellulose, pectin, phytohormones, and chlorophylls. The upstream reasons should be diverse and complicated, in this study we first detected the changes of shikimate-related pathways, because shikimate is directly upstream to the lignin pathway which is the major target influenced by *BnMYB69* downregulation.

**Figure 8:**
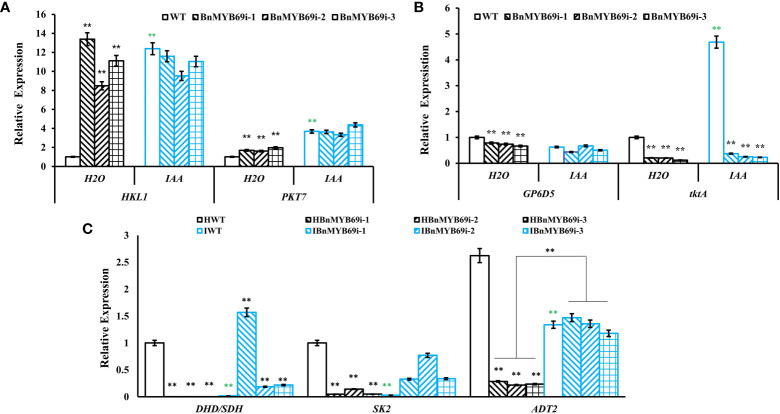
Expression of key genes of related metabolic pathways under different treatments. **(A)** Expression of key genes of glycolysis pathway in different materials. **(B)** Expression of key genes of pentose phosphate pathway in different materials. **(C)** Expression of *DHD/SDH, SK2* and *ADT2* in shikimate pathway in different materials. Asterisks * or ** indicate significant or extremely significant differences from HWT (*0.01<p < 0.05; **p< 0.01) using one-way ANOVA.

qRT-PCR showed that the expression of key genes of the glycolytic pathway (*HKL1* and *PKT7*) was significantly upregulated in transgenic plants, and IAA treatment completely mitigated this effect (P < 0.01) (**Figure 8A**). The expression of key genes of the pentose phosphate pathway (*GP6D5* and *tktA*) (**Figure 8B**) and shikimic acid pathway (*ADT*2) were significantly downregulated in *BnMYB69i* plants (P < 0.01) (**Figure 8C**). IAA treatment completely recovered expression levels of *ADT*2 and *GP6D5*, over-compensated the expression of *DHD/SDH* and *SK2*, but had little recovery on *tktA* (**Figure 8C**). These results show that the pentose phosphate pathway and shikimic acid pathway, in coordination with the upstream glucose metabolic pathway, are most likely to be regulated by *BnMYB69* together with IAA signaling, and different loci have differed responses.

**Figure 9:**
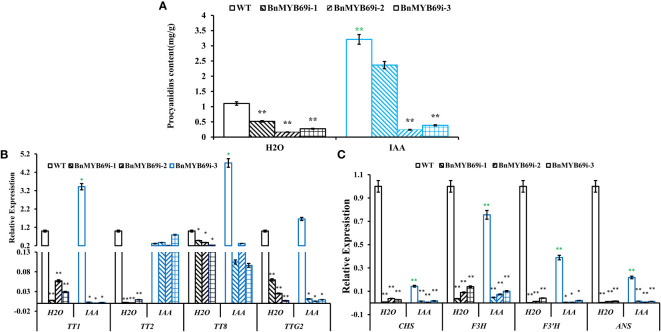
Changes of proanthocyanidin (PA) synthesis genes in transgenic and WT plants under different treatments. **(A)** Changes of PA content under different treatments. **(B, C)** Expression of PA synthesis related genes of different materials under different treatments. Asterisks indicate significant or extremely significant differences from WT (*0.01≤ p < 0.05; **p< 0.01) using one-way ANOVA.

Downstream the shikimate pathway, flavonoid-proanthocyanidin (PA) pathway is neighboring to lignin pathway and these two pathways have competition in metabolic flux. Thus, we detected the influence of *BnMYB69* downregulation on the flavonoid-PA pathway. Like lignin content, the PA content in the leaves of *BnMYB69i* plants was also significantly lower than WT, and IAA treatment exerted a little compensation (**Figure 9A**). It was confirmed that shikimates, lignins, and PAs are all decreased by *BnMYB69* downregulation.

The expression of key genes involved in PA biosynthesis was also analyzed using qRT-PCR (**Figures 9B, C**). In *BnMYB69i* plants, expression levels of core flavonoid biosynthesis genes *CHS*, *F3H* and *F3’H*, anthocyanin biosynthesis gene *ANS*, and PA pathway regulatory genes *TT1*, *TT2*, TT8 and *TTG2* were all significantly lower than those of WT plants (P < 0.05). IAA treatment did not exert recovery effect on most of these loci, but *TT2* expression was completely recovered. These results showed that *BnMYB69* regulated the shikimate-flavonoid-PA compound pathway, independent of or downstream IAA signaling, but IAA is involved in *BnMYB69* regulation on *TT2* expression.

## Discussion

### BnMYB69 is a S21 R2R3-type transcription factor

The R2R3-MYB family is the largest and most widely studied family of MYB transcription factors. In addition to the similar functions of 1R-MYB family members, the members of this family are widely involved in plant life processes such as primary metabolism and biological and abiotic stress responses (Ito, 2005). Researchers believe that most members of the R2R3 MYB protein family are involved in regulating plant-specific physiological and biochemical processes such as plant secondary metabolism and cell morphogenesis (Wan and Li, 2002). In addition, many members of the R2R3 MYB gene family isolated from different species regulate anthocyanin biosynthesis (Czemmel et al., 2009; Machado et al., 2009; Feng et al., 2010; Lin-Wang et al., 2011). However, most *MYB* transcription factors have been cloned, analyzed and studied in *Arabidopsis*, some researchers have found that MYB TFs play vital roles in plant development and anthocyanin metabolism, and *PAP1/2* can promote the expression of anthocyanin biosynthesis genes (Muleke et al., 2021). In fact, MYB transcription factor members play a role in regulating growth and development in different parts of different time periods. For example, *AtMYB115* and *AtMYB188/PGA37* participate in regulating the seed germination rate at the seed germination stage (Wang et al., 2009); *AtMYB30*, *AtMYB38*, and *AtMYB18/LAF1* regulate hypocotyl elongation (Yang et al., 2009; Froidure et al., 2010); and *AtMYB21*, *AtMYB24*, *AtMYB26*, *ATMYB35*, *AtMYB57*, *GhMYB80*, and *AtMYB99* participate in anther development (Millar and Gubler, 2005; Xu et al., 2014). There are also some MYB transcription factors, such as *BnMYB43* and *GbMYBR1*, involved in lateral organ development and stem morphogenesis, which regulate the number of plant branches and the development of plant higher organs (Jiang et al., 2020; Su et al., 2020).

In this study, four *BnMYB69* genes were isolated, characterized, and functionally elucidated. Results of systemic analysis on the structure and homology features of their genes and proteins all indicated that they encoded a S21 R2R3-MYB transcription factor, orthologous to AtMYB69. There are few reports on the function of MYB69. Previous studies prove that AtMYB69 is a transcriptional activator dedicated to secondary cell wall thickening of fiber cells (Dubos et al., 2010). According to other reports on the function of MYB transcription factors similar to ATMYB69 protein, these transcription factors are not only related to secondary wall synthesis (Zhong et al., 2008; Kim et al., 2012), but also related to anthocyanin metabolism (Peng et al., 2019; Xu et al., 2021), plant resistance (Xu et al., 2020; Im et al., 2021), etc. Therefore, systematic study of the function of *MYB69* is helpful to providing insight into the biofunction of *MYB* gene family.

### Silencing of *BnMYB69* affected lodging resistance and *S. sclerotiorum* resistance

The *MYB69* transcription factor is related to xylem formation and is strongly expressed in stems. Many researchers have found that the expression of key genes in lignin synthesis is related to lodging traits, particularly *COMT* (Ma, 2009; Li et al., 2013), *F5H* (Li and Qi, 2011; Li et al., 2013), *4CL* (Li and Qi, 2011; Huang et al., 2013; Li et al., 2013) and *PAL* (Huang et al., 2013). Our results showed that silencing *BnMYB69* reduced the lodging resistance of rapeseed. Cytological observation of cross-sections and longitudinal sections of stems and the expression of key genes of lignin metabolism in transgenic plants revealed a positive correlation with lignin synthesis. This shows that BnMYB69 is an activator of lignin synthesis in *B. napus*.

In addition to lignin, plant cell wall components also contain cellulose and pectin. Cellulose and pectin content analysis showed that the cell wall of *BnMYB69i* plants developed poorly. This might be the reason that the resistance to *S. sclerotiorum* in *BnMYB69i* plants was significantly decreased compared with that in WT plants.

The effect of *BnMYB69*s on roots is quite different from or even opposite to its effect on stems, particularly on monolignol types. The proportion of S/G in *BnMYB69i* plants increases in stems and decreases in roots, which may contribute to the different physical characteristics of the roots and the stems. Furthermore, lignin, cellulose and pectin increased in roots, though decreased in the stems, indicating that there was a significant difference between the aboveground and underground parts of *BnMYB69i* plants. Since the basic difference between aboveground and underground is dark or in light, this phenomenon implies that *BnMYB69i* functioning depends on light condition.

### *BnMYB69* silencing leads to changes in the cell cycle, resulting in plant dwarfing

The offspring of *BnMYB69i* were generally shorter than those of WT plants. Pith cells and unit volume were greater based on electron microscopy and staining, and the expression of most cell cycle-related genes in the stems of transgenic plants was significantly lower than that in the stems of WT plants. This indicates that cell division in transgenic plants was not vigorous and rapid, and the total number of cells should be reduced, which was consistent with the results of cells in the previous cross-cut stems. In addition, the IAA content in the stems of transgenic plants was lower than that of WT plants, which confirmed that plant cell division was not vigorous. Furthermore, the increase in ABA content led to plant dwarfing and root development changes. Antisense transgenic plants and overexpression plants of the rice dwarf gene *OsDWARF48* were analyzed, and the IAA content of the dwarf plants was lower than that of WT plants (Li et al., 2018).

qRT-PCR results confirmed that auxin can also promote cell division by degrading the expression of CDK inhibitor protein Kip-related proteins (KRPs). The statistical results of the number of cells (**Figure 4B**) in the stems of transgenic plants also show that the change in cell division caused a change in the number of xylem cells and then caused a change in stem height and stem circumference. Since *BnMYB69* is mainly expressed in tissues under lignification, thus it is logical that the confinement of *BnMYB69* downregulation on cell cycle and cell division is mainly on the secondary growth and plant height, and the promotion effect on parenchyma cells, lateral growth and total biomass might be because of more flux branch-off from suppressed secondary metabolism.

### *BnMYB69* silencing affects IAA synthesis, photosynthetic reaction, and glucose metabolism

The results of the hormone content tests on the stems and leaves of the offspring of transgenic plants showed that the content of IAA was significantly lower than that of WT, indicating that *BnMYB69* affected the synthesis of IAA. The precursor of IAA is tryptophan, which regulates the growth, development and resistance of plants. Researchers have found that expression of the *BnMYB193* gene in *B. napus* was also induced by IAA, ABA and other hormones to promote the development of lateral roots of rapeseed and improve the lodging resistance of plants (Wu et al., 2018).

In the IAA compensation experiment, *BnMYB69* affected the shikimic acid pathway upstream of IAA synthesis. In addition, the pentose phosphate pathway upstream of the shikimic acid pathway was also affected by *BnMYB69*. It can be inferred that *BnMYB69* has regulatory functions on metabolic pathways since sugar metabolism, although sugar synthesis is affected by photosynthesis. The up and down regulation of the hexokinase gene in plants leads to extensive plant phenotypic changes such as overexpression of *AtHKL1* in tomato, inhibition of plant growth, reduction of chlorophyll content and photosynthetic rate in leaves, and photochemical quantum efficiency reduction (Dai et al., 1999). Inhibiting *AtG6PD5* expression causes plants to have short stems and leaves, curly leaves and light leaf color (Wang, 2013). *BnMYB69i* transgenic plants indeed had lower plant height, which also confirmed the regulatory function of *BnMYB69*.

In a study of *B. napus*, *TT1* gene silencing led to the downregulation of flavonoid pathway genes such as *TT3*, *TT4*, *TT5*, *TT6*, *TT7* and *TT18*. The interference of *BrTT1* gene expression in yellow seed *B. napus* causes the abnormal or absent expression of several important *BrTT1* genes in the flavonoid biosynthesis pathway, which then blocks the accumulation of PAs resulting in a transparent seed coat. The *BnTT1* gene in *B. napus* also regulates the PA and flavonoid metabolism pathways (Lian et al., 2017).

*ANS* is the most critical gene in the anthocyanin biosynthesis pathway. *UFGT* and *ANS* cooperate to transform unstable anthocyanins into stable anthocyanins in the cytoplasm and vacuole. Among the five genes of the anthocyanin biosynthesis pathway, only *ANS* and *DFR* are regulated by *TTG2*. Studies on tobacco show that *TTG2* and *ARF8* work together to control the degree of flower color by regulating the expression of *ANS* and *DFR* in the anthocyanin biosynthesis pathway (Li et al., 2017). *ARF8* relies on *TTG2* to regulate the expression of *ANS* and *DFR*, which affects anthocyanin accumulation. *TTG2* inhibition prevents plant trichome formation, anthocyanin accumulation and seed color pigmentation. Similarly, *AtTT8* is also involved in the regulation of anthocyanin and PA biosynthesis in *Arabidopsis* (Baudry et al., 2006; Xu et al., 2013). Complementation analysis in *Arabidopsis TT8* mutant showed that *NnTT8* in *Nelumbo nucifera* could function similarly to *AtTT8* in regulating anthocyanin and proanthocyanin biosynthesis (Deng et al., 2021). The downregulated expression of *TT1*, *TTG2, TT8, ANS* and *DFR* in *BnMYB69i* transgenic plants also confirmed its impact on flavonoid compounds and the anthocyanin synthesis pathway, which explains the increase in surface trichomes of transgenic plants.

### Chlorophyll synthesis in *BnMYB69*-silenced plants was more sensitive to light intensity

The entire process of chlorophyll synthesis requires 15 enzymatic reactions. Among them, *HEMA* is the coding gene that catalyzes ALA synthesis. The *HEMA* family has three members in *Arabidopsis*, and the expression of *HEMA1* is induced by light (McCormac et al., 2001), whereas *HEMA2* and *HEMA3* are not induced by light, and the *HEMA3* gene is not even expressed. However, the *HEMA1* gene is expressed in all plant tissues, and its expression is higher in photosynthetic tissues (Tanaka et al., 1996). CHLM and CRD can catalyze the transformation of protoporphyrin IX into chlorophyll ester a, and knock out of *CRD* in *Arabidopsi*s resulted in slow plant growth and development and chlorosis of leaves (Bang et al., 2008). CHLG acts to form chlorophyll a, and CAO catalyzes Chla oxidation to form Chlb. Under strong light, chlorophyll synthesis increases, and the PSII reaction intensifies, such that the expression of the two coding genes increases. The *PORA* gene, a member of the *POR* family, is a coding gene for protochlorophyll ester oxidoreductase. The *PORA* gene has a photoinduction effect, and it is also the main enzyme of plant leaf chlorophyll accumulation and the only confirmed gene in the phytochrome A-mediated mechanism for the greening blocks (Sperling et al., 1997). Some researchers have shown that *PORA* overexpression is sufficient to guide the synthesis of a large amount of chlorophyll and the development of normal plants in the absence of *PORB* and *PORC* (Paddock et al., 2010). However, the expression of *PORA* decreased in that experiment. It was speculated that *PORA* was affected by the *BnMYB69* gene, and the expressions of *PORB* and *PORC* may be increased. Mechanism of BnMYB69 effect on chlorophyll synthesis and light reaction will be deeply studied in the future.

## Data availability statement

The original contributions presented in the study are included in the article/**Supplementary Material**. Further inquiries can be directed to the corresponding author.

## Author contributions

YC: Designing the research, analyzing some data, and revising the manuscript. NL: Designing and doing some experiments, analyzing most data, and writing the original manuscript. MW and JJ: Doing some experiments. QZ and JY: Providing writing suggestions. JLi: Providing some plant material. JLian and YX: Providing some technologies of the experiment. All authors: reading this manuscript and agreeing with publication. All authors contributed to the article and approved the submitted version.
