# Supplementary material for: Downregulation of Brassica napus MYB69 (BnMYB69) increases biomass growth and disease susceptibility via remodeling phytohormone, chlorophyll, shikimate and lignin levels
Source: Front Plant Sci. 2023 Mar 29;14:1157836. doi: 10.3389/fpls.2023.1157836 (PMC10108680; doi:10.3389/fpls.2023.1157836)
Supplement: Supplementary file 1 [file DataSheet_1.docx]

Downregulation of *Brassica napus MYB69* (*BnMYB69*) Increases Biomass Growth and Disease Susceptibility via Remodeling Phytohormone, Chlorophyll, Shikimate and Lignin Levels

**Supplementary Material**

| **TABLE S1 PCR primers for cloning, expression, subcellular localization, and RNAi studies of *BnMYB69*s** | | |
| --- | --- | --- |
| **Primer name** | **Primer sequence（5’——3’）** | **Purpose** |
| FBMYB69-31 | GCTCATCGGATCCAGGGGAATCGTTG | 3’RACE gene-specific primers |
| FBMYB69-32 | ACGGCCACTTCTACGTTCAACCAAA |  |
| RBMYB69-51 | TGAGTTCTTCTTATGATCTGGTGTAGCTG | 5’RACE gene-specific primers |
| RBMYB69-52 | GGTGCTGAAACAAAAGTGTAAGGCCAG |  |
| FBnMYB69-1Q | GGATACAAGTCGTGGTCACTG | *BnMYB69-1* qRT-PCR |
| RBnMYB69-1Q | GAAAGCAACATTTCTCTTCCCACC |  |
| FBnMYB69-2Q | GGACAAGAGCCATGGTACCTC | *BnMYB69-2* qRT-PCR |
| RBnMYB69-2Q | GAAAGCAACATTTCTCTTCCCACC |  |
| FBnMYB69-3Q | ATTGCCCGATTATTCCCTGGGC | *BnMYB69-3* qRT-PCR |
| RBnMYB69-3Q | AGAGCTTTCACGTTTGCGTCTAGCT |  |
| FBnMYB69-4Q | CATTGCCCGATTATTCCCTGGGA | *BnMYB69-4* qRT-PCR |
| RBnMYB69-4Q | AGAGCTTTCACGTTTGCGTCTTGCC |  |
| FBnMYB69-1 | ATTCCAATTTATTTTTCTTTCTTTTGTCTTGT | *BnMYB69-1* full length |
| RBnMYB69-1 | GGACGGAAAATGAGAGTTGTAATTG |  |
| FBnMYB69-2 | AGACTAATCCATCATATTCCTCTTTC | *BnMYB69-2* full length |
| RBnMYB69-2 | GACGGTCTAATGTAACTAACGCA |  |
| FBnMYB69-3 | GATGTAGGTGGAGATAGAGATTG | *BnMYB69-3* full length |
| RBnMYB69-3 | ATCATATAAAACATGCTTTCTAATACAAGC |  |
| FBnMYB69-4 | GATGTAGGTGGAGATAGAGATTG | *BnMYB69-4* full length |
| RBnMYB69-4 | ATAAAATTAGTAGACTTGCATATATCCTTG |  |
| M13F | CGCCAGGGTTTTCCCAGTCACGAC | Cloning vector detection and sequencing |
| M13R | AGCGGATAACAATTTCACACAGGA |  |
| FBnMYB69SL | GAATTCATGGAAATGTCTAGAGGAAGCAAT | *BnMYB69* subcellular localization |
| RBnMYB69SL | GGATCCCTAAGAAAGTAATCCAACTCCAAG |  |
| FBnMYB69I | GGATCCGACGTCAGACGCAAACGTGAAAGCTTA | *BnMYB69* RNA interference |
| RBnMYB69I | TCTAGACCATGGCTAAGAAACTAATCCAACTCCAAG |  |
| F35S3N | GGAAGTTCATTTCATTTGGAGAG | RNAi vector construction and detection, RNAi transgenic plants detection |
| FBnPAP2I2 | ATTTAAATGACGTCAGGTTTACATTCAAGACACA |  |
| RBnPAP2I2 | CCTAGGTGGATTCGTACGTAAACTTTT |  |
| ROCST5N | GCTCAGGTTTTTTACAACGTGCAC |  |
| UPM | CTAATACGACTCACTATAGGGC | 5’-RACE universal primers |
| NUP | AAGCAGTGGT AACAACGCAGAGT |  |
| 3’P | GCTGTCAACGATACGCTACGTAACG | 3’-RACE universal primers |
| 3’NP | CGCTACGTAACGGCATGACAGTG |  |

| **TABLE S2 Structure and homology features of the four *BnMYB69* genes** | | | | | | | | | |
| --- | --- | --- | --- | --- | --- | --- | --- | --- | --- |
| **Gene name** | **gene length (bp)** | **Intron number** | **mRNA length (bp)** | **ORF length (bp)** | **5’UTR length (bp)** | **Transcription start sites (TSS)** | **3’UTR length (bp)** | **Polyadenylation sites** | **Gene/mRNA/ORF identities with *AtMYB69* (%)** |
| *BnMYB69-1* | 1110 | 1 | 1015 | 756 | 132 | A_1_, A_110_ | 127 | C_1068_, C_1110_ | 84.2/85.5/86.5 |
| *BnMYB69-2* | 1418 | 1 | 1320 | 753 | 73 | A_1_, A_14_, A_51_, G_55_, G_88_, G_138_ | 494 | C_1051_, T_1059_, T_1076_, T_1078_, T_1080_, T_1096_, T_1175_, T_1401_, C_1418_ | 78.3/80.0/86.2 |
| *BnMYB69-3* | 1296 | 1 | 1191 | 732 | 28 | G_1_ | 431 | T_1016_, T_1095_, T_1120_, C_1170_, T_1296_ | 77.8/79.5/84.8 |
| *BnMYB69-4* | 1215 | 1 | 1110 | 756 | 28 | G_1_, G_10_, T_15_, A_18_ | 326 | T_1089_, T_1166_, T_1215_ | 80.0/80.8/87.0 |

According to cloning results, nucleotide sequences of *BnMYB69*s were created, annotated, and analyzed on Vector NTI Advance 11.51.

| **TABLE S3 Various features of the four BnMYB69 proteins** | | | | | | | | | | |
| --- | --- | --- | --- | --- | --- | --- | --- | --- | --- | --- |
| **Protein name** | **Identities/positives with AtMYB69 (%)** | **Length (aa)** | **MW (kDa)** | ***pI*** | **Phosphorylation sites** | **Signal peptide** | **Subcellular localization** | **Transmembrane helix** | **HTH MYB-type domain region** | **H-T-H motif DNA-binding region** |
| BnMYB69-1 | 82.5/85.7 | 251 | 29.01 | 9.58 | 19S, 8T, 1Y | no | nuclear | 0 | 14-65, 66-120 | 42-63, 93-116 |
| BnMYB69-2 | 82.0/85.2 | 250 | 28.90 | 9.70 | 20S, 8T, 2Y | no | nuclear | 0 | 14-65, 66-120 | 42-63, 93-116 |
| BnMYB69-3 | 80.9/84.1 | 243 | 28.16 | 9.72 | 22S, 9T, 1Y | no | nuclear | 0 | 14-64, 66-120 | 42-63, 93-116 |
| BnMYB69-4 | 82.9/85.7 | 251 | 29.04 | 9.56 | 21S, 9T, 1Y | no | nuclear | 0 | 14-65, 66-120 | 42-63, 93-116 |

Protein sequences of BnMYB69s were created according to gene annotation and analyzed for length, MW and *pI* on Vector NTI Advance 11.51. Other protein features were analyzed on websites: phosphorylation sites by NetPhos 3.1 (https://services.healthtech.dtu.dk/service.php?NetPhos-3.1), signal peptide by SignalP-5.0 (https://services.healthtech.dtu.dk/service.php?SignalP-5.0), subcellular localization by Plant-mSubP (http://bioinfo.usu.edu/Plant-mSubP/), transmembrane helix by DeepTMHMM (https://dtu.biolib.com/DeepTMHMM), conserved domain/motif search at ScanProsite (https://prosite.expasy.org/).

BnMYB69
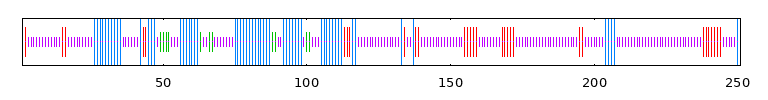
-1 (α-helix 23.11%, extended strand 12.75%, β-turn 4.38%, random coil 59.76%)


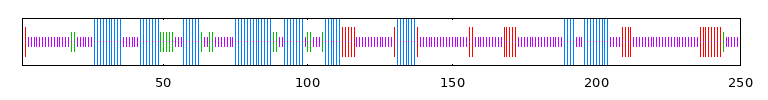
BnMYB69-2 (α-helix 27.60%, extended strand 11.60%, β-turn 6.40%, random coil 54.40%)


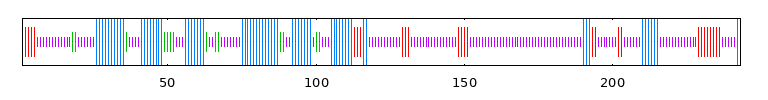
BnMYB69-3 (α-helix 26.75%, extended strand 10.70%, β-turn 6.17%, random coil 56.38%)


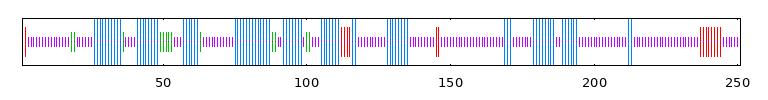
BnMYB69-4 (α-helix 31.87%, extended strand 6.77%, β-turn 5.18%, random coil 56.18%)

**FIGURE S1 SOPMA-predicted secondary structures of BnMYB69s proteins**

https://npsa-prabi.ibcp.fr/cgi-bin/secpred_sopma.pl

BnMYB69-1 BnMYB69-2 BnMYB69-3 BnMYB69-4

**FIGURE S2 SWISS-MODEL-predicted tertiary structures of BnMYB69s proteins**

https://swissmodel.expasy.org/

**FIGURE S3 Expression organ-specificity of individual member genes of *BnMYB69*s**

Note, 1, hypocotyl; 2, cotyledon; 3, root; 4, stem; 5, leaf; 6, flower; 7, pod peel; 8, seed. All values are presented as the mean ±standard deviation (SD). Asterisks indicate significant or extremely significant differences from the first series (*, 0.01≤p < 0.05; **, p< 0.01) using one-way ANOVA (the same below)


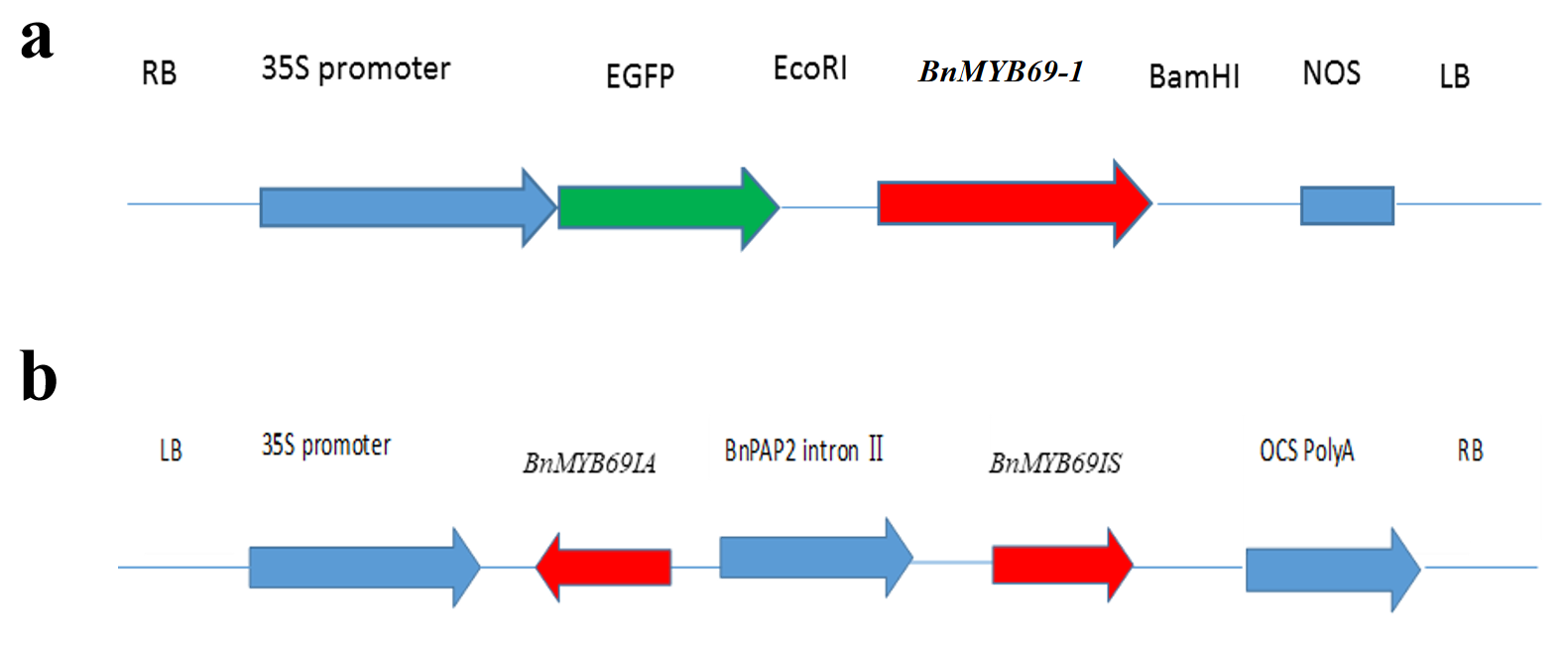


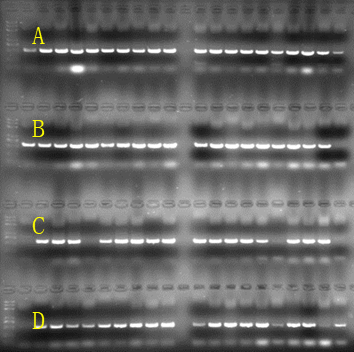


**c**

**FIGURE S4 Vector construction charts and transgenic plants detection of *BnMYB69-1***
